# Using comics in teaching mathematics to improve junior high school students’ disaster awareness

**DOI:** 10.4102/jamba.v15i1.1345

**Published:** 2023-01-27

**Authors:** Mailizar Mailizar, Rahmah Johar, Yulinar Safitri, Sulastri Sulastri, Siti Fatimah, Ulya Rohaizati

**Affiliations:** 1 Department of Mathematics Education, Universitas Syiah Kuala, Banda Aceh, Indonesia; 2 Department of Chemistry Education, Universitas Syiah Kuala, Banda Aceh, Indonesia; 3 Realistic Mathematics Education Research Centre, Universitas Syiah Kuala, Banda Aceh, Indonesia

**Keywords:** mathematical comics, disaster education, disaster awareness, urban students, rural students

## Abstract

Students’ awareness of disasters is vital, especially for those living in disaster-prone areas, so it needs to be developed during the learning. One strategy to increase students’ awareness is integrating the concept of disaster awareness in learning, such as using mathematical comics based on disaster contexts. This study aimed to examine the improvement of junior high school students’ awareness of disasters through mathematical comics. It also compared the improvement of awareness of the urban and rural students. This study employed a quantitative approach with a pre-test–post-test control group design. The population was all Year 7 students in one of the junior high schools in Banda Aceh (urban area) and one of the junior high schools in Aceh Besar (rural area), Indonesia. The samples were of two classes (experimental and control) in each school, which are selected randomly. Data were collected through a disaster awareness questionnaire and analysed by *t*-test.

**Contribution:**

This study showed that using mathematics comics in different schools with varying conditions resulted in the different levels of disaster awareness-raising. Students’ disaster awareness in the urban experimental class was better on three of the four indicators, namely, pre-disaster awareness, false disaster awareness, and after disaster awareness, compared to the control class. Meanwhile, the rural experimental class did better on one of the four indicators, namely, disaster education awareness, than the control class. This study implies that comics can increase disaster awareness and the need for further research.

## Introduction

Disasters are natural- and human-caused events leading to the loss of life, property and social values and can disrupt human activities (Özey 2006; Şahin & Sipahioğlu 2007). Disasters are generally triggered by either natural conditions or human activities. Indonesia, a country where three tectonic plates meet (Australia, Eurasia and the Pacific), is prone to disasters. Indonesia ranks first in terms of tsunami hazards. Disasters in Indonesia have caused the loss of more than 200 000 of lives, for example, the Aceh earthquake and tsunami in 2004 (BNPB 2021). Aceh is one of the provinces in Indonesia that is highly vulnerable to disasters. Almost every year, disasters, such as earthquakes, floods, landslides and droughts, occur in Aceh (BNPB 2018). Aceh also has a long history of previous major disasters, such as the earthquake and tsunami in 2004 and the earthquake in Pidie Jaya in 2016, resulting in significant economic losses.

Increasing disaster awareness is part of the preparations to deal with disasters. The increased disaster awareness will lead to improved disaster protection and prevention behaviour, which will minimise the threat of disasters (Özgüven & Öztürk 2006). Disaster awareness is not limited to the action during or after a disaster but preventive action and preparations before a disaster. Thus, disaster awareness needs to be disseminated through educational practices (Johnson et al. 2014). It can be realised through the dynamic and continuous involvement of all relevant parties (Khorram-Manesh et al. 2016; Wang 2016).

In general, student disaster awareness is lacking (Ahmad et al. 2017; Pinar 2017), despite that the damage because of disaster can be reduced by increasing disaster awareness, which can be done systematically (Clerveaux, Spence & Katada 2010). In addition, natural disasters hit not only in rural areas but also in urban areas. Therefore, efforts are needed to improve disaster awareness for students in rural and urban areas, including integrating disaster awareness education into mathematics learning.

Mathematics learning delivered to all students should be adapted to their needs in each school (Wijaya 2012). Appropriate learning strategies are necessary to increase students’ awareness of disasters. Mathematical comics with a disaster context as a learning medium is one solution to help students understand mathematics. Comics is one of the fiction or non-fiction media presented through visual images consisting of fine art cartoons designed to create humour when conveying moral messages (Toh et al. 2016). However, it is hard to find comics relevant to mathematics learning, and limited studies have been conducted on comics for mathematics learning (Toh et al. 2017).

The visual arts of comics provided in text and images enhance and expand the communication to facilitate readers to create their understanding (McVicker 2007). In addition, visuals also help readers develop their imaginations in real contexts (Pratt 2009). Meanwhile, the context in a broad sense refers to everyday life phenomena, fictional or fantasy stories, or also mathematical problems that occurred directly (Wijaya 2012). In other words, contextual problems or everyday life situations are used to construct and apply mathematical concepts.

Mathematical comics contain various information to raise awareness of disasters for their readers. Mathematical comics with disaster contexts provide useful information about disasters for students. It presents the condition of Aceh 17 years ago when the earthquake and tsunami hit and post-disaster conditions. The information includes an understanding of what a disaster is, types of disasters, natural and non-natural disasters, what to do in the event of a disaster, types of earthquakes, and the earthquakes that occurred in Aceh, the causes of frequent small earthquakes, contacts in the event of a disaster, predictable disasters, earthquake and tsunami simulations, what to do in case of an indoor earthquake, signs for evacuation directions, the need for contacting family or relatives to convey post-disaster conditions, checking electricity and gas if at home during a disaster to avoid other disasters such as fires, family preparations to deal with disasters, organisations in charge of disaster management, the impact of disasters and a safe place to live to be safe from disasters, such as tsunami.

A study on students’ awareness of disasters was done by Ahmad et al. (2017) and Pinar (2017), revealing that student awareness of disasters was generally lacking. Sarwidi (2018) examined disaster awareness and showed that disaster preparedness education could be conducted in schools by fostering awareness of disaster hazards and risks. Nuzulidar (2020) also researched the development of math problems in a disaster context. However, research on mathematical comics to increase students’ awareness of disasters is limited, especially in comparing urban and rural students. Therefore, it is important to research the increase in student disaster awareness, focusing on comparing urban and rural students. The research problem of this study is: Is there a significant difference in the increase of disaster awareness between urban and rural students who are taught using mathematics comics?

## Literature review

### Urban and rural schools

Education is critical for everyone, and schools play a role in it. The government builds schools in urban and rural areas to ensure that everyone has access to education. However, in general, schools in rural areas are affected by various negative factors impacting the education quality. They are usually remote and relatively underdeveloped. As a result, many schools lack supporting resources to achieve maximum learning goals (Mulford & Johns 2004; Peters & Le Cornu 2004). In addition, students studying in urban areas have different parental socioeconomic status (SES) from those in rural areas. The low SES of parents in rural areas disadvantages the students. Parents in rural areas are usually not concerned with school. They also cannot afford to buy additional items needed for learning, which negatively impacts on teaching and learning in schools.

All students (rural or urban) must obtain a quality education to promote educational progress in rural areas. Rural students find it difficult to engage in education because of the low quality of education facilities, resulting in lower achievement (Taylor & Mulhall 2001). However, most solutions depend on adequately trained, motivated and engaged teachers in rural areas. In addition, students in rural schools, especially in remote rural ones, may be limited by the narrow curriculum coverage in their schools (Monk 2007; Oakes & Maday 2009) and limited practicum activities, hindering students’ opportunities to develop education (Howley, Rhodes & Beall 2009).

Many factors lead to lower educational participation in rural compared to urban schools. Firstly, students in rural areas are usually less interested in attending school. Also, students in urban areas have higher motivation and willingness to attend school than in rural areas (Lockheed & Verspoor 1991). In addition, many rural parents depend on their children for help with work, while schools are usually designed to follow rigid schedules, meaning less time to help parents (Taylor & Mulhall 2001).

Secondly, parents’ education level in urban areas is overall different from that of rural areas. Rural parents often have lower levels of education and may lack positive views of schools. It may be because of the perceived lack of school relevance worsened by the rigid curricula, which often designed for contexts (and sometimes cultures) unrelated to rural areas. Rural schools rarely adapt curricula to use local examples or link curricula to local needs (Taylor & Mulhall 2001).

Thirdly, rural parents are less capable of helping their children in learning. They are not well-educated and thus have a lower ability to support their children. Furthermore, houses in rural areas do not have facilities for learning (Taylor & Mulhall 2001). In short, children in rural areas may find it more challenging to obtain an education. They tend to have less parental encouragement to go to school and more demands on their free time, such as helping parents at work. In addition, they may find the curriculum less relevant to their lives and less supportive of their learning at home.

### Disaster context

Disaster is a condition disrupting the community functions, including individual, material, economic losses and impacts that the community cannot overcome. Immadudina (2011) argued that a disaster is an event in nature because of humans or nature that can harm human life, disrupt normal life and cause the loss of property and objects. Coppola (2015) stated that disasters occur because of natural and non-natural factors, leading to loss of life and damaging infrastructure. It can be concluded that a disaster is an event or a series of events caused by natural factors or the surrounding human behaviour, resulting in losses.

There are 13 types of disasters caused by natural and non-natural factors. Disasters because of natural factors include earthquakes, tsunamis, volcanic eruptions, floods, landslides, droughts and hurricanes. Meanwhile, disasters because of non-natural factors are bushfires, technological failures, disease outbreaks, social conflicts and transport accidents (BNPB 2020).

Law No. 24 of 2007 concerning disaster management defines a disaster as an event or series of events that threaten and disrupt people’s lives and livelihoods because of natural, non-natural and human factors, resulting in environmental damage, loss of property and psychological impacts. The law clearly states that everyone has the right to get education, training, counseling and skills in disaster management in situations where there is no disaster or a situation where there is a potential for disaster. Through education, it is hoped that disaster risk reduction efforts can achieve broader targets and be introduced earlier to all students by integrating disaster risk reduction education into the school curriculum and extracurricular activities.

*Minister of Home Affairs Decree of Republic of Indonesia* No. 131 of 2003 states that mitigation is an effort and activity conducted to reduce and minimise the consequences of disasters, including preparedness, vigilance and various skills to overcome them. Meanwhile, Prasad (2010) argued that disaster mitigation includes actions that reduce the severity of future disasters. It includes structural mitigation actions (such as developments in city regulations and building ethics) and non-structural mitigation actions (such as implementing school safety programmes and community awareness programmes).

Disaster management can be interpreted as a process consisting of several phases: the pre-disaster stages, namely, preparation, emergency response and post-disaster or rehabilitation. Each component involved will intertwine. Similarly, Shaluf (2007) asserted that disaster management involved all aspects of planning to respond to disasters, referring to manage disaster risks and consequences. Meanwhile, disaster management includes prediction, warning, emergency assistance, rehabilitation and reconstruction (Moe & Pathranarakul 2006).

In addition, Moe and Pathranarakul (2006) also make a life cycle of disaster management and modern disaster management, consisting of four important characteristics in disaster management: mitigation, preparedness, response and recovery. Mitigation is an action taken before a disaster to reduce disaster risk or eliminate the impact of a disaster. Meanwhile, preparedness is a plan to respond to a disaster, also defined as a state of readiness to face crises, disasters and other emergency conditions. While the response is the immediate action taken before, during and after a disaster event to save lives, reduce damage, and improve early disaster recovery. Furthermore, recovery is an activity to return the infrastructure system to a minimum operational standard and the guideline of long-term efforts designed to return life to normal or a better condition.

The risk analysis based on the Guidelines for Disaster Risk Mitigation Planning developed by the Indonesian National Disaster Management Agency (*Regulation of the Head of BNPB Number 4 of 2008*) is done using the following formula:


$$R=\frac{H\times V}{C}$$ [Eqn 1]

where:

*R* = disaster risk; *H* = hazard threat (the frequency or possibility of certain disasters tends to occur with a certain intensity at certain locations); *V* = vulnerability (expected loss or impact in a given area in case of a certain disaster occurs with a certain intensity. The calculation of this variable is usually defined as the exposure [population, assets, etc.] times of the sensitivity for the specific intensity of the disaster); C = adaptive capacity (the capacity available in the area to recover from a particular disaster).

Potential disaster hazards are unavoidable, but the damage can be reduced, and people’s ability to deal with disasters can be increased. Based on the previous formula, integrating disaster context in mathematics learning increases the capacity, which reduces disaster risk. When the capacity is greater, the disaster risk will be lower. The components affecting the magnitude of the impact of a disaster are threats, vulnerabilities and capacities.

**FIGURE 1:**
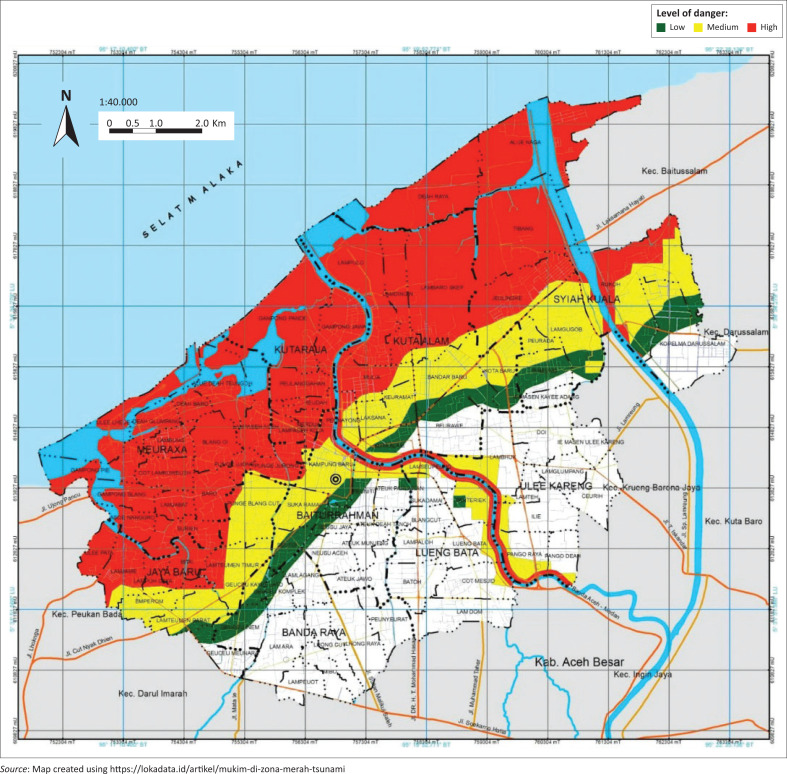
Location of the data collection.

Aceh is one of the provinces in Indonesia affected by the tsunami in 2004, so disaster education is paramount to be implemented in schools, especially in disaster-prone areas. Figure 1 shows a map of the tsunami disaster district in Aceh. In Aceh, the Disaster Preparedness School (or *Sekolah Siaga Bencana* – SSB) was established in 2009, integrating disaster education into the school curriculum to improve disaster preparedness. The Disaster Preparedness Schools have integrated the context of disaster into the subjects of Religion, Bahasa, Social Sciences, Natural Sciences, local content and extracurricular activities carried out outside school hours (Adiyoso 2018). Thus, the disaster context can also be integrated into mathematics learning, which can reduce disaster risk.

### Learning mathematics with disaster context

Education is a systematic effort to realise the learning and learning atmosphere, so that students actively develop their potential to be spiritual and religious, as well as have self-control, intelligence, excellent personality, noble character and skills needed by students, society and nation. Education is also defined as one of the primary needs of humans. With education, humans will have a clearer and more focused mindset and life direction. Therefore, excellent education should not only prepare students for a profession but also solve problems in everyday life and apply knowledge in any conditions.

Learning is one of the means of education achieved through a directed process (Santrock 2007). Learning is an interaction between students, teachers and learning resources in a learning environment, where the centre of these activities is the students. Mathematics learning has an important role in preparing students for future social life (Gravemeijer et al. 2017).

Learning mathematics is where the teacher allows students to construct their knowledge according to mathematical principles. Learning mathematics is not just delivering information from teachers to students; instead, it aims to equip students to use reasoning and logic in solving complex mathematical problems (Wilson, Fernandez & Hadaway 1989). This mathematics learning will run well when students use their real-life experiences to build their knowledge.

Mathematics learning should be adapted to the needs of students in each school (Wijaya 2012). It is expected to help students to understand the learning and make learning more meaningful. Learning models or strategies are expected to help students to understand mathematics. One of them is learning mathematics applying a disaster context. Students living and studying in disaster-prone locations need learning that integrates the disaster context. In addition, media can be used to facilitate integration.

### Comics as learning media

The mathematical comics used in this study present mathematical problems and information about disasters. The problems use a disaster context and ratio. The disaster information used is about marine ecosystems, such as Acropora coral reefs, mangrove forest planting to reduce the impact of disasters and disaster volunteers needed when a disaster occurs. In addition, mathematical problems also discuss what to do if an earthquake occurs and someone is on a bridge. It also covers selecting the right route and the scale on the map to get to the evacuation site. The problems presented in mathematical comics use ratio, one of Year 7 topics. The problems also contain indicators of logical–mathematical intelligence.

Mathematical comics with disaster contexts provide useful information about disasters for students. It presents the condition of Aceh 17 years ago when the earthquake and tsunami occurred and the post-disaster conditions. Mathematical comics contain various information to raise awareness of disasters for their readers. The information includes: (1) understanding a disaster; (2) types of disasters; (3) disasters caused by natural and non-natural factors; (4) what should be done in the event of a disaster; (5) what kinds of earthquakes and earthquakes have occurred in Aceh; (6) frequent causes of small earthquakes; (7) contacts in the event of a disaster; (8) predictable disaster; (9) earthquake and tsunami simulation; (10) what to do in case of an indoor earthquake; (11) evacuation direction signs; (12) the need to contact family or relatives to convey the post-disaster conditions; (13) checking electricity and gas if at home during a disaster, so that it does not cause other disasters, such as fire; (14) family preparations to deal with disasters; (15) organisations in charge of disaster management; (16) the impact of the disaster; and (17) a safe place to live to be safe from disasters, such as tsunamis.

### Disaster awareness

The process of adopting a new behaviour, according to Rogers (Notoatmodjo 2014), occurs through (1) awareness, the person is aware in the sense of knowing the stimulus (object); (2) interest, one begins to be interested in the stimulus; and (3) evaluation, considering whether or not the stimulus is good for him/her; (4) trial, one starts to try new behaviour; and (5) adoption, one has behaved in a new way based on his/her knowledge, awareness and attitude towards the stimulus. If the acceptance or adoption of a new behaviour occurs based on awareness, knowledge and a positive attitude, the behaviour will be long-lasting; otherwise, it will be temporary.

Awareness is one’s ability to think, will, and feel (Soekanto 2012). Awareness is part of the ability to refuse to do anything that does not benefit him/her. Awareness integrates knowledge into attitudes and manifests in daily behaviour or actions (Neolaka 2008).

Soekanto (2012) proposed four hierarchical components of awareness, from the lowest to the highest: knowledge, understanding, attitudes and behaviour patterns (actions). Atkinson, Atkinson and Hilgard (1997) stated that awareness includes perceptions, thoughts and feelings. In addition, the theory of conscientisation (awareness) adds knowledge, attitudes and regulations as the components of awareness.

Disaster awareness is the condition of a person with knowledge, understanding, skills and concern with disaster matters, so that he or she has the awareness to behave and adapt in disaster-prone areas well and can actively participate in minimising the occurrence of disasters, disaster or overcome the impact in the event of a disaster. Disaster awareness is not only limited to take action during or after a disaster but also to take necessary precautions and preparation before a disaster. These measures are at least as effective as the response to disasters. Risk reduction and pre-disaster preparation activities are the most important stages of modern disaster management.

Individual behaviour in dealing with disasters is in line with the level of preparedness and awareness. The potential hazards of disasters cannot be prevented, but the damage can be reduced, and the human capacity to deal with disasters can be improved. Public awareness of disasters must be improved to achieve this goal. Disaster awareness should be increased to develop disaster protection and prevention behaviour, thereby minimising disaster risk (Özgüven & Öztürk 2006). People with disaster awareness have sufficient information about disasters. They may be aware of the potential threats and risks and, consequently, can create action plans for disasters and act appropriately during disasters.

Disaster experiences encourage disaster awareness (Hoffmann & Muttarak 2017). Disasters and emergencies have a devastating impact on students globally. Unfortunately, they suffer the most from natural disasters and lack the knowledge of how to protect themselves in such situations. In particular, educating and raising students’ awareness should be one of the common and fundamental problems. It is recognised that providing disaster education and fostering disaster awareness to preschool students is critical. Strengthening disaster awareness and disaster preparedness will be achieved primarily through education. Therefore, the full integration of disaster education across the curriculum is paramount.

## Methods

### Research design

This study employed a quantitative method with a pre-test–post-test control group design (Creswell 2014). Two groups were selected randomly, and a pre-test was given to determine the initial ability between the experimental and the control groups:


$$\begin{array}{llll} \text{Eksperiment group}: & O_{1} & X & O_{2}\\ \text{Control group }: & O_{1} & & O_{2} \end{array}$$ [Eqn 2]

where:

*O*_*1*_ = pre-test; *X* = learning using mathematical comics; *O*_*2*_ = post-test.

### Population and sample

The population was Year 7 students of one of the junior high schools in Banda Aceh and one of the junior high schools in Aceh Besar, Indonesia. The school is located in a disaster-prone area and is a Disaster Preparedness School (SSB). The junior high school in Banda Aceh is the school, while the junior high school in Aceh Besar is a rural school. Data were collected in the area hit by the 2004 Indian Ocean tsunami (see red area in the map).

The samples were randomly selected (four classes): two experimental and two control classes. Each school consisted of experimental and control classes.

### Research instrument

The instrument used in this research was a disaster awareness questionnaire, adopted from Dikmenli, Yakar and Konca (2018), which had been tested for reliability and validity. The questionnaire consisted of 36 items meeting indicators of Disaster Education Awareness, Pre-Disaster Awareness, False Disaster Awareness and After Disaster Awareness. This questionnaire used a 5-point Likert scale, ranging from ‘Strongly Disagree’ to ‘Strongly Agree’ (SA). The disaster awareness questionnaires were administered before the learning took place and at the end of the lesson. The student awareness questionnaire on the disaster was translated, validated and proofread by a linguist. After being proofread, it was tested for its readability on Year 7 students. The readability test results showed that the questionnaire was feasible to use, and the students understood the meaning of the items in the questionnaire (see Appendix 1 for the scale items).

### Design and its use in learning

**FIGURE 2:**
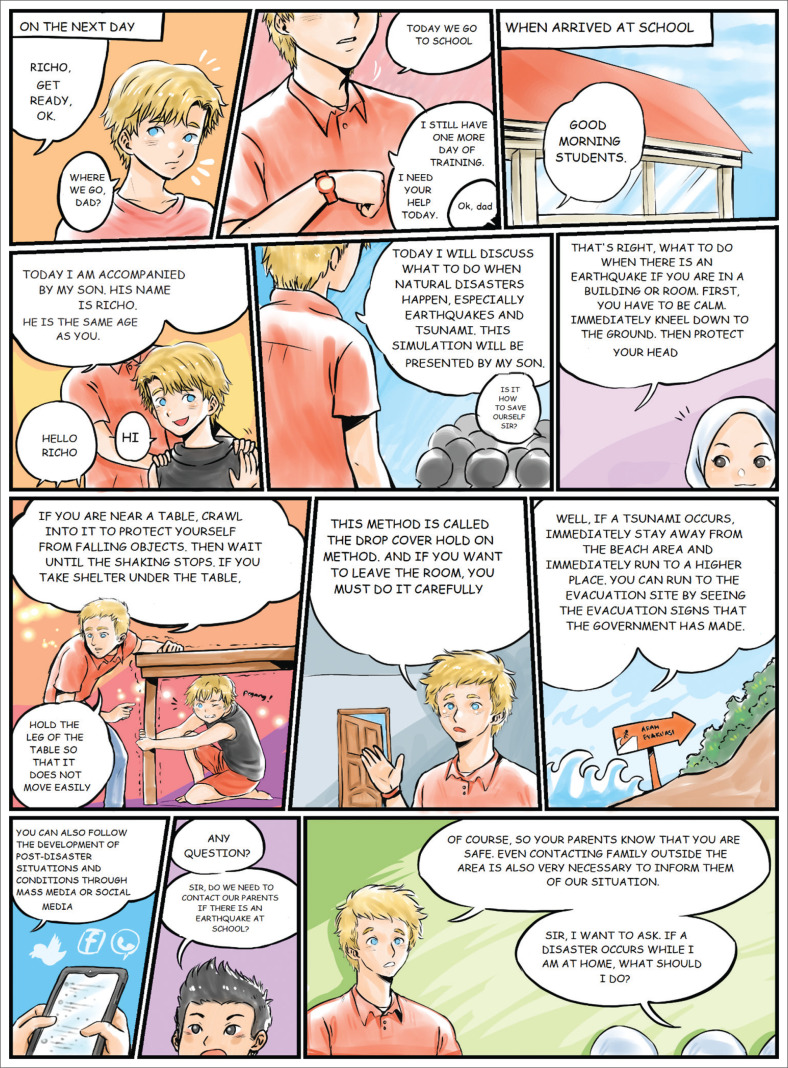
An example of information presented in the comics (translated to English).

The mathematical comics used a disaster context and were designed to improve disaster awareness, especially for junior high school students. It presents various information about the earthquake and tsunami disasters and what to do if such a disaster occurs. Figure 2 shows an example of the information about disasters presented in the comics (translated to English).

Students read the comics at home and during their learning at school. It illustrates what to do when a disaster occurs. The teacher also explains some information about the disaster, so that students understand better.

### Data collection procedure

The disaster awareness data in this study were obtained through pre-test and post-test carried out in both the experimental and control classes. The learning was done in three lessons and two lessons for pre-test and post-test. In the experimental class, before the learning began, students were given mathematical comics to read at home because of limited time available at school. During the learning, the comic was only read briefly.

### Data analysis

The data obtained from the disaster awareness questionnaire were analysed quantitatively. Data analysis was done as follows: (1) transforming the questionnaire data from ordinal to interval scale using method successive interval; (2) calculating pre-test, post-test, and gain scores; and (3) calculating the magnitude of the increase in disaster awareness from the pre-test and post-test scores using the normalised gain formula (N-gain). Table 1 shows the gain index criteria based on Hake (1999).

**TABLE 1:** N-gain score criteria.

N-gain score	Interpretation
*0.70 < g*	High
0.30 ≤ *g* ≤ 0.70	Moderate
*g* < 0.30	Low

*Source:* Adapted from Hake, R.R., 1999, *Analyzing change/gain score*, viewed n.d., from http://www.physics.indian.edu/nsdi/AnalyzingChange-Gain.pdf

In this study, questionnaires were administered before and after all teaching and learning activities ended. The analysis of research questions or items was calculated for each question or item. It was done to examine a significant difference in the increase in disaster awareness of urban and rural students. In addition, an analysis of indicator questions or items was also done to evaluate which indicators of disaster awareness could be achieved by urban and rural students.

### Ethical considerations

Approval to conduct the study was obtained from the Institution of Research and Community Service, Universitas Syiah Kuala, reference number: 341/UN11/SPK/PNBP/2021.

## Results and discussion

### Disaster awareness average and N-gain

Data on student disaster awareness were obtained from the disaster awareness questionnaires given before (pre-test) and after the learning (post-test) in the experimental and control classes. The data were first transformed from ordinal data to the interval. The average of pre-test and post-test can be seen in Table 2.

**TABLE 2:** Disaster awareness score data.

Schools	Score	Experiment		Control	
		*N*	$\bar{X}$	*N*	$\bar{X}$
Urban schools	Pre-test	27	101.30	27	103.26
	Post-test	27	105.89	27	106.45
Rural schools	Pre-test	34	98.25	34	104.03
	Post-test	34	96.00	34	107.21

Maximum ideal score = 180.

Table 2 depicts that the average pre-test of disaster awareness of urban students in the experimental and control classes is 101.30 and 103.26, respectively. Meanwhile, it is 98.25 and 104.03 for the experimental and control classes in the rural school. This finding indicates that the pre-test scores in both the experimental and control classes in the two schools were relatively equal. The average post-test score in the experimental and control classes in the urban school is 105.89 and 106.45, respectively. Meanwhile, it is 96.00 and 107.21 for experimental and control classes in the rural schools. This shows that the pre-test and post-test scores of students’ disaster awareness in urban and rural schools are different.

Table 3 presents the N-gain of students’ awareness of the experimental and control class disasters.

**TABLE 3:** N-gain score data on student disaster awareness.

Schools	Experiment				Control			
	*N*	N-gain	Mean	Criteria	*N*	N-gain	Mean	Criteria
Urban schools	27	0.97	0.05	Low	27	1.44	0.05	Low
Rural schools	34	−1.14	−0.03	Low	34	−2.34	−0.07	Low

Maximum ideal score = 180.

Table 3 indicates that, in the rural school, the experimental class students using mathematical comics overall have a higher total N-gain than the control class. Meanwhile, in the urban school, the N-gain of the control class is higher than of the experimental class. The total N-gain score of students in the experimental and control class in the urban area was 0.97 and 1.44, respectively. Meanwhile, the total N-gain score of the experimental and control classes in the rural school is -0.03 and -2.34, respectively.

### Disaster awareness of each indicator

Furthermore, in this study, students’ disaster awarness was assessed based on four indicators of disaster awareness, namely, disaster education awareness, pre-disaster awareness, false disaster awareness, and after disaster awareness. We presented data of students’ responses from experimental and control groups based on location of schools (rural and urban). Table 4 shows the responses of students from experimental groups at urban schools.

**TABLE 4:** Percentage of urban students’ responses from experimental groups.

Indicator	Experimental group									
	Pre					Post				
	Strongly disagree	Disagree	*N*	Agree	Strongly agree	Strongly disagree	Disagree	*N*	Agree	Strongly agree
Disaster Education Awareness	0	0	22.2	70.4	7.4	0	0	25.9	70.4	3.7
Pre-Disaster Awareness	0	0	3.7	74.1	22.2	0	0	25.9	59.3	14.8
False Disaster Awareness	0	0	63.0	29.6	7.4	0	7.4	48.2	40.7	3.7
After Disaster Awareness	0	0	14.8	59.3	25.9	0	0	40.7	51.9	7.4

Table 4 shows that most students in the experimental group at the urban school responded agree in both the pre-test and post-test. However, two indicators significantly change their response between the pre-test and post-test. In the first one, for the indicator of pre-disaster awareness, 74.1% of students agreed, while in the post-test, only 59.3% of students remained with the same opinion. On the other hand, there was a difference in pre- and post-tests for false disaster awareness. In the pre-test, 29.6% of students responded agree, while 40.7% of students have this opinion in the post.

In Table 4, the student responded to Strongly Disagree and Disagree at 0%. This incident naturally occurred because students considered that disaster education was important. For example, item 14 in questionnaire ‘planning and preparation should be done with family members for disasters’. This activity is very important for students who live in disaster areas.

Furthemore, Table 5 shows the responses of students in the urban school from the control group.

**TABLE 5:** Percentage of urban students’ responses from control groups.

Indicator	Control group									
	Pre					Post				
	Strongly disagree	Disagree	*N*	Agree	Strongly agree	Strongly disagree	Disagree	*N*	Agree	Strongly agree
Disaster Education Awareness	0	0	14.8	81.5	3.7	0	3.7	29.6	66.7	0
Pre-Disaster Awareness	0	0	7.4	81.5	11.1	0	7.4	22.2	40.7	29.6
False Disaster Awareness	0	18.5	44.4	37.1	0	3,7	22.2	51.9	22.2	0
After Disaster Awareness	0	0	14.8	81.5	3.7	0	3.7	22.2	74.1	0

Table 5 shows that most students agreed to all indicators in the control group in both the pre-test and post-test. On one hand, in the pre-test, there were no students who responded Strongly Agree. Interestingly, in the indicator of pre-disaster awareness, there is a significant shift in students’ responses to Strongly Agree. On the other hand, in the post-test, 29.6% of students strongly agreed with this indicator. However, for the other indicator, the results show that the percentage of positive responses (Agree and Strongly Agree) tends to be lower in the post-test than in the pre-test.

On the other hand, in rural schools, students’ responses tend to be more positive in post-test than in pre-test. The results show that three out of four indicators had a significant change to Strongly Agree.

Table 6 shows that rural school students in the experimental class who responded to the questionnaire with Strongly Agree in the pre-test experienced an increase in the post-test in two indicators. It reveals that during the pre-test, the percentage for the pre-disaster awareness indicator increased from 23.5% to 52.9% and false disaster awareness from 2.9% to 82.4%.

**TABLE 6:** Percentage of rural students’ responses from experimental groups.

Indicator	Experiment group									
	Pre					Post				
	Strongly disagree	Disagree	*N*	Agree	Strongly agree	Strongly disagree	Disagree	*N*	Agree	Strongly agree
Disaster Education Awareness	0	0	2.9	97.1	-	0	0	0	94.1	5.9
Pre-Disaster Awareness	0	0	2.9	73.5	23.5	0	0	0	47.1	52.9
False Disaster Awareness	0	0	38.2	58.8	2.9	0	0	0	17.6	82.4
After Disaster Awareness	0	0	14.7	58.8	26.5	0	0	26.5	73.5	0

Regarding the control group in the rural school, Table 7 shows that rural school students who responded to the questionnaire with Strongly Agree in the pre-test experienced a decrease in post-test in three indicators. However, the after disaster awareness has increased from 0% to 8.8%.

**TABLE 7:** Percentage of rural students’ responses from control groups.

Indicator	Control group									
	Pre					Post				
	Strongly disagree	Disagree	*N*	Agree	Strongly agree	Strongly disagree	Disagree	*N*	Agree	Strongly agree
Disaster Education Awareness	0	0	11.8	88.2	0	0	0	2.9	97.1	0
Pre-Disaster Awareness	0	0	0	88.2	11.8	0	0	2.9	91.2	5.9
False Disaster Awareness	0	8.8	61.8	26.5	2.9	0	8.8	20.6	70.6	0
After Disaster Awareness	0	0	32.4	67.6	0	0	0	2.9	88.2	8.8

Overall, results show that the average score of student disaster awareness on each indicator differs between urban and rural schools. Table 8 presents the average disaster awareness scores across urban and rural schools.

**TABLE 8:** Average of student disaster awareness score on the rural school each indicator.

School	Class	Indicator			
		Disaster education awareness	Pre-disaster awareness	False disaster awareness	After disaster awareness
Urban school	**Experiment**				
	Pre-test	80.49	77.96	74.88	75.03
	Post-test	77.38	81.74	77.62	77.64
	Difference	−3.11	3.78	2.74	2.61
	**Control**				
	Pre-test	77.89	79.81	72.08	80.04
	Post-test	81.13	82.76	74.00	80.77
	Difference	3.24	2.95	1.92	0.73
Rural school	**Experiment**				
	Pre-test	89.49	82.02	94.41	107.10
	Post-test	92.28	81.71	93.21	94.99
	Difference	2.79	−0.31	−1.2	−12.11
	**Control**				
	Pre-test	100.88	107.02	93.06	104.30
	Post-test	98.36	102.74	93.93	97.83
	Difference	−2.52	−4.28	0.87	−6.47

Table 9 reveals that the experimental class students in the urban school performed better on the indicators of pre-disaster awareness, false disaster awareness and after disaster awareness compared to the control class. The results show that for students in the experimental group, there were improvements in the pre-disaster awareness (3.78), false disaster awareness (2.74) and after disaster awareness (2.61). On the other hand, for the control class, there were improvements in the pre-disaster awareness indicator (32.95), false disaster awareness indicator (1.92) and after-disaster awareness (0.73). Meanwhile, the experimental class students in rural schools performed better on the indicators of disaster education awareness than in the control class.

**TABLE 9:** Results of *t*-test for the *N*-gain score of student disaster awareness.

Schools	*t*-test for equality of means			Conclusion
	*f*	*df*	Sig.	
Urban schools	0.420	52	0.198	Not significant
Rural schools	3.036	66	0.353	Not significant

The results presented in Table 9 indicate no significant difference between the N-gain score of rural and urban schools (*p* > 0.05). In other words, the difference in the increase in student disaster awareness of students taught using mathematical comics and those without is not significant in both urban and rural schools.

## Discussion

This study examines the increase in the disaster awareness of junior high school students using mathematical comics and compares the increase between students in urban and rural schools. In this study, from urban schools, 27 students participated in the experimental group, and 27 students were involved in the control group. For the rural school, 34 students participated in the experimental group and 34 in the control group. Overall, participants of the study have positive responses towards using mathematics comics in their learning activities. Based on the study’s findings, there are three important findings to discuss.

Firstly, not all indicators of disaster awareness in urban and rural schools experienced an increase, both in the experimental and control classes. Secondly, the increase in disaster awareness of students in urban and rural schools shows that no significant difference between students taught using mathematical comics and those without. It can be seen from the pre-test and post-test scores in urban and rural areas. Thirdly, the average of N-gain in the experimental and control classes in both urban and rural schools is low.

The findings may be because of the long gap between lessons. Schools in rural areas had the first, second, and third lessons on 27 May 2021, 28 May 2021 and 14 June 2021, respectively. Meanwhile, in urban areas, the first, second and third lessons were on 22 April 2021, 28 April 2021 and 11 June 2021, respectively.

In addition, this research was conducted during the coronavirus disease 2019 (COVID-19) pandemic. The rapid spread of the COVID-19 virus disrupted this research, and it was stopped temporarily because of school closures. Students were suddenly required to take semester exams during the school closure, and the research resumed after the semester. The topic in this study was also an enrichment topic, so that it could continue after the exam.

The time spent during the learning affects students’ awareness. Awareness is the process of changing one’s behaviour, and it takes a long time to grow it. Notoatmodjo (2014) believed that changing or adopting new behaviour is a complex process and requires a relatively long time.

Changes in the learning system also disrupted students’ learning concentration. This is in line with Winata’s research (2021) regarding the focus of student learning during the pandemic, revealing that the students’ focus during the pandemic is low, especially during the shifting of learning methods. One’s concentration can be influenced by many factors, including the learning environment. Novianti (2019) argued that the environment plays an important role in one’s concentration level. Quiet environmental conditions positively influence student concentration while studying. It also affects the calmness of students to study and disturbs their psyche. Psychological factors of students, such as the ability to think, emotions, beliefs and self-scheme, interests and motivation, have a big influence in creating positive mathematics learning conditions to achieve learning objectives (Nurdiana 2017).

During a pandemic, students’ attention was often divided. Many students are concerned about the learning method applied during the pandemic, influencing students learning and, ultimately, student disaster awareness. Atkinson et al. (1997) asserted that psychological factors such as thoughts and feelings affect a person’s awareness. Students were also more focused on mathematics problems than the information presented in comics about disasters, so that information was sometimes ignored. Students were required to read more comics at home than during learning. Furthermore, the teacher did not strengthen the disaster information in the comics at the end of the lesson. The teacher only reinforced the group discussion results related to mathematical problems. Teachers were accustomed to cognitive strengthening, so they missed strengthening the affective aspects. This reinforcement is necessary, because students read comics at home without the teacher’s supervision. Some students did not read comics thoroughly and got little information about disaster awareness.

## Conclusion

This study concludes that using mathematical comics in different schools with different conditions results in different disaster awareness. The N-gain of students’ disaster awareness in urban and rural schools is low, and no significant between students taught using mathematical comics and those without. The use of mathematics comics in urban schools can increase disaster awareness on indicators of pre-disaster awareness, false disaster awareness, and after disaster awareness. However, it cannot increase the indicator of disaster education awareness. While in rural schools, the use of mathematics comics can increase disaster awareness on the indicator of the disaster education awareness. However, the use of comics cannot increase indicators of pre-disaster awareness, false disaster awareness and after-disaster awareness. This finding indicates that rural and urban schools have different characteristics of students. Therefore, using the same treatment, which is integrating mathematics comics to increase students’ disaster awareness, does not produce the same results. Therefore, it is necessary to conduct further studies to teach rural students better to increase their disaster awareness.

## Appendix 1

**TABLE 1-A1:** Disaster Awareness Scale.

Number	Description
**Disaster education awareness**	
1.	I need to tell my parents and relatives that I am safe after a disaster.
2.	I think first aid training is important in disaster education.
3.	Disasters can happen anywhere, anytime.
4.	The destructive effects of disasters should be shown to students.
5.	I think I know the disaster types in general.
6.	I can define disaster.
7.	Potential risks that may cause a disaster should be determined in advance.
8.	I am aware that a disaster may happen any time.
9.	I think that while the buildings are a great risk for some disasters, they may provide great protection against some disasters.
10.	Nuclear and chemical accidents are actually technological disasters.
11.	I find the scope of disaster education given in schools inadequate.
12.	I find it unnecessary to set a meeting point during disasters.
13.	I think that I need some general information about disasters.
**Pre-disaster awareness**	
14.	Planning and preparation should be done with family members for disasters.
15.	It is best to follow the warnings that the authorities will make from various communication means such as speakers and radio.
16.	I think societies should be organised against disasters.
17.	It is very important to identify a contact person from outside of a disaster region to ensure communication after a disaster.
18.	Disaster and Emergency kits should be available in every house.
19.	I mostly know disaster types that cause material damage and loss of life.
20.	Some of the disasters’ arrival times can be predicted beforehand.
21.	I am aware of the risky disasters in my region.
**False disaster awareness**	
22.	It is unnecessary to act cautiously if it is my destiny to be hurt in a disaster.
23.	I see it as a waste of time applying the ‘drop-cover-hold on’ method during disasters.
24.	It is not possible to reduce destructive effects of disasters.
25.	The concept of natural disaster reminds me only of earthquakes.
26.	Disaster education is not necessary for everyone.
27.	It is more accurate to talk about the consequences of disasters rather than the measures to be taken.
28.	If it is in your destiny, you cannot escape from disasters.
29.	Shutting down electricity, water and natural gas vents at the time of evacuation is very time consuming.
**After disaster awareness**	
30.	I think I am good enough about applying first aid after a disaster.
31.	I know which government agency to contact after a disaster.
32.	I know how I will be informed during emergencies and where to stay.
33.	I know which numbers to call in case of disasters.
34.	I must be prepared enough to deal with disasters.
35.	I know the after disaster ‘meeting point’ in my neighbourhood.
36.	I am aware of the organisations that are working to reduce the damage of disasters.
